# Supplementary material for: Liver Kinase B1—A Potential Therapeutic Target in Hormone-Sensitive Breast Cancer in Older Women
Source: Cancers (Basel). 2019 Jan 28;11(2):149. doi: 10.3390/cancers11020149 (PMC6406422; doi:10.3390/cancers11020149)
Supplement: Supplementary file 1 [file cancers-11-00149-s001.pdf]

Supplementary Materials: Liver Kinase B1—A Potential Therapeutic Target in Hormone-Sensitive Breast Cancer in Older Women

Binafsha Manzoor Syed, Andrew R Green, David A L Morgan, Ian O Ellis and Kwok-Leung Cheung

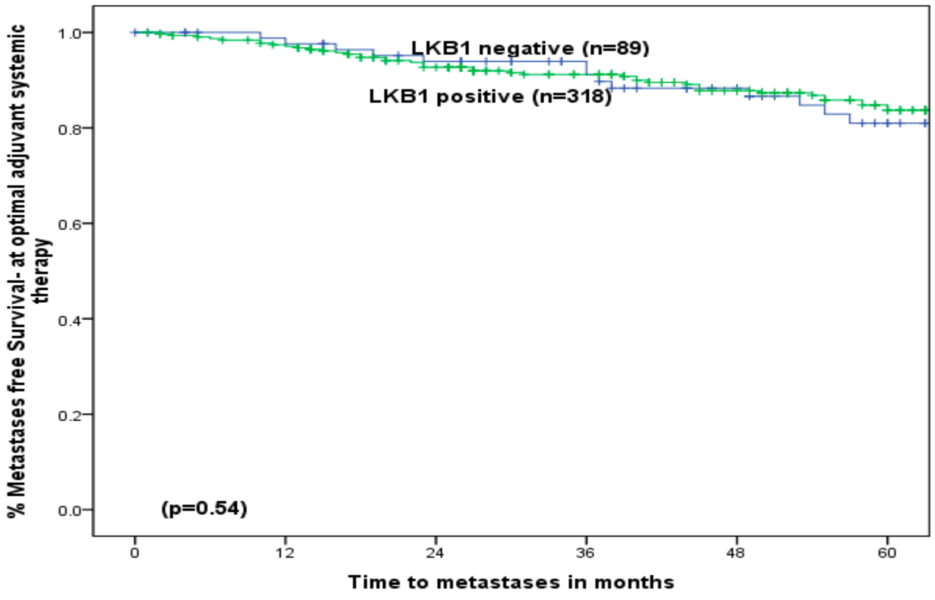

(a)

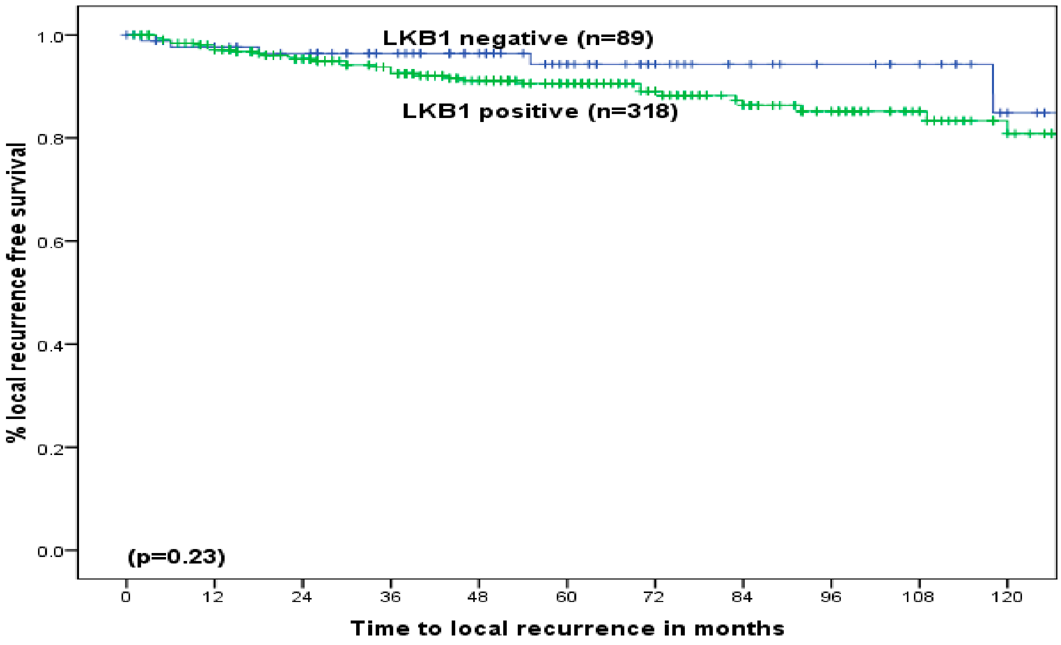

(b)

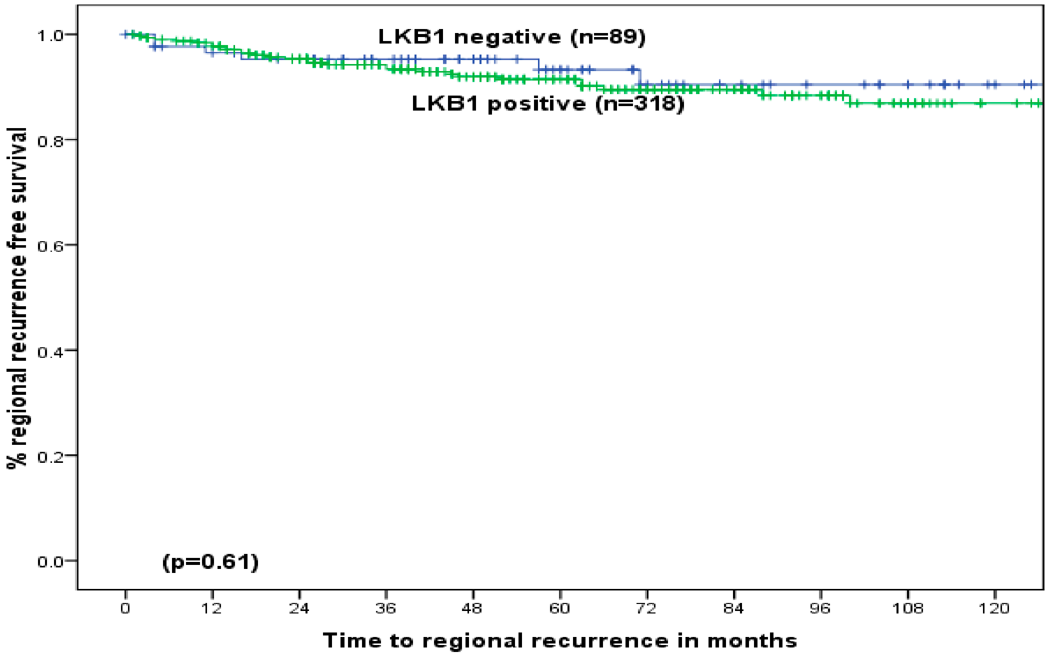

(**c**)

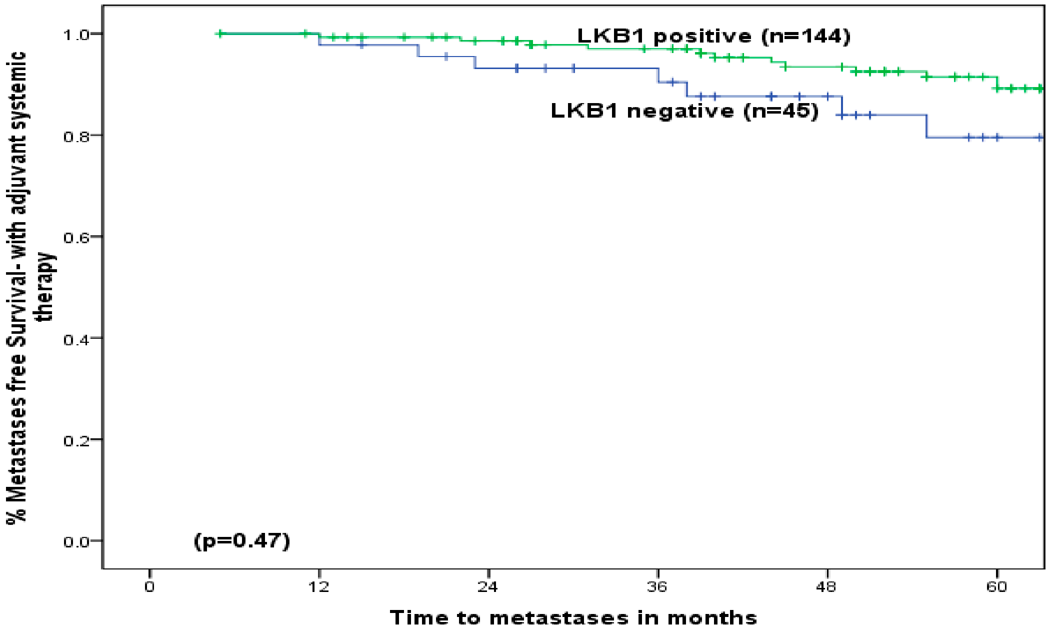

(**d**)

Figure S1. (**a**) Metastasis-free survival according to the expression of LKB1 in older women with early operable primary breast cancer (all patients). (**b**) Local recurrence-free survival according to the expression of LKB1 in older women with early operable primary breast cancer (all patients). (**c**) Regional recurrence free survival according to the expression of LKB1 in older women with early operable primary breast cancer (all patients). (**d**) Metastasis-free survival according to the expression of LKB1 in older women with early operable primary breast cancer who received adjuvant endocrine therapy.

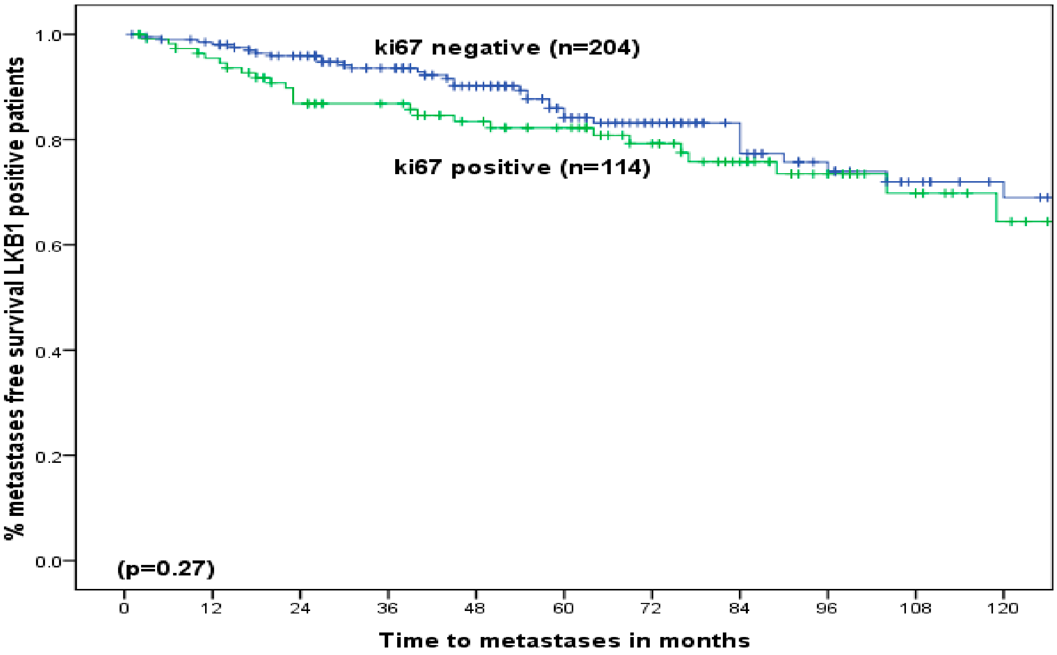
ki67 negative (n=204)
ki67 positive (n=114)
(p=0.27)
% metastases free survival LKB1 positive patients
1.0
0.8
0.6
0.4
0.2
0.0
0
12
24
36
48
60
72
84
96
108
120
Time to metastases in months

(a)

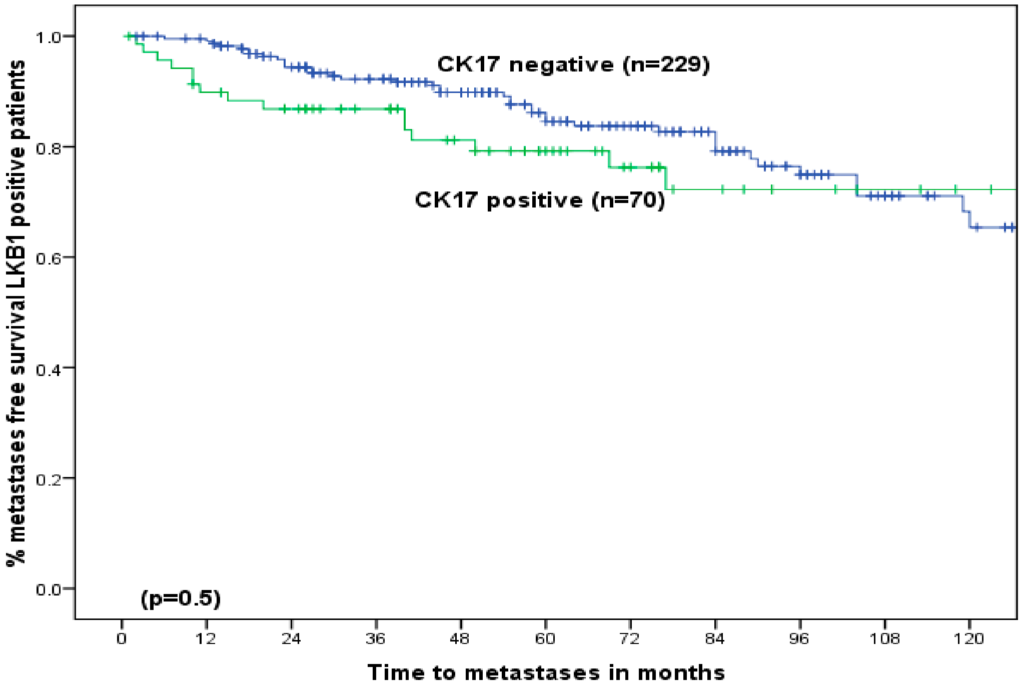
CK17 negative (n=229)
CK17 positive (n=70)
(p=0.5)
% metastases free survival LKB1 positive patients
1.0
0.8
0.6
0.4
0.2
0.0
0
12
24
36
48
60
72
84
96
108
120
Time to metastases in months

(b)

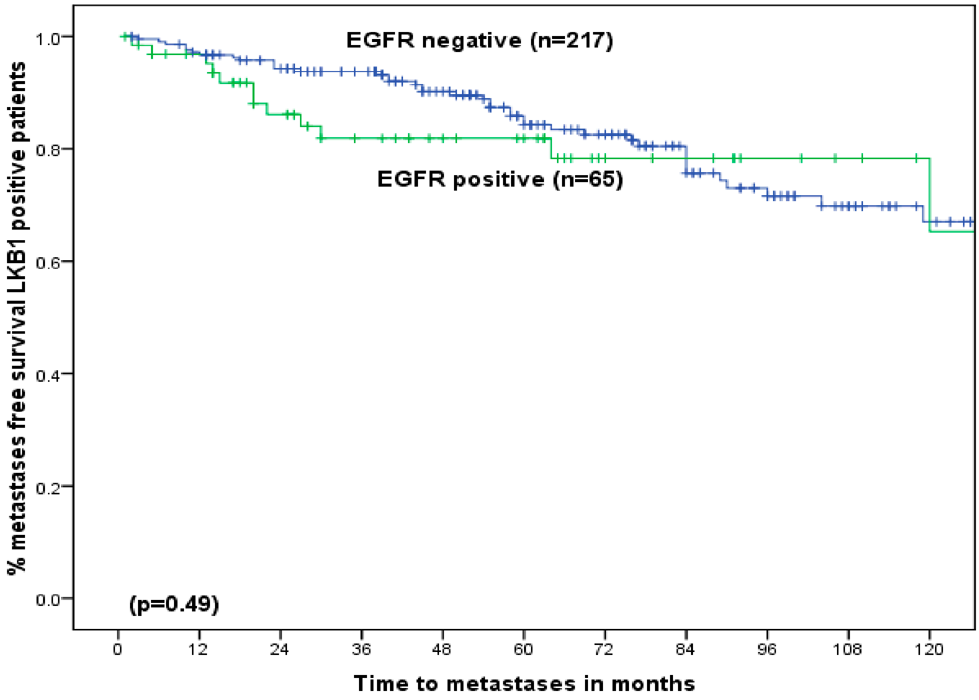

(**c**)

Figure S2. (**a**) Metastases free survival in LKB1 positive early operable primary breast cancer in older women: Ki67 positive versus Ki67 negative. (**b**) Metastasis-free survival in LKB1 positive early operable primary breast cancer in older women: CK17 positive versus CK17 negative. (**c**) Metastasis-free survival in LKB1 positive early operable primary breast cancer in older women: EGFR positive versus EGFR negative.

© 2019 by the authors. Submitted for possible open access publication under the terms and conditions of the Creative Commons Attribution (CC BY) license (http://creativecommons.org/licenses/by/4.0/).
